# Advances in Smart Materials and Structures

**DOI:** 10.3390/ma16227206

**Published:** 2023-11-17

**Authors:** Bing Wang, Tung Lik Lee, Yang Qin

**Affiliations:** 1 Fujian Provincial Key Laboratory of Terahertz Functional Devices and Intelligent Sensing, School of Mechanical Engineering and Automation, Fuzhou University, Fuzhou 350108, China; 2 ISIS Neutron and Muon Source, STFC Rutherford Appleton Laboratory, Harwell Campus, Didcot OX11 0QX, UK; tung-lik.lee@stfc.ac.uk; 3 School of Civil Aviation, Northwestern Polytechnical University, Xi’an 710072, China; yang_qin@nwpu.edu.cn

Smart materials and structures are capable of active or passive changes in terms of shapes (geometries), properties, and mechanical or electromagnetic responses, in reaction to an external stimulus, such as light, temperature, stress, moisture, and electric or magnetic fields. They have attracted increasing interest for their enhanced performance and efficiency over a wide range of industrial applications, especially in the field of aerospace. These applications require novel engineering approaches and design philosophy in order to integrate the actions of sensors, actuators, and control circuit elements into a single system that can respond adaptively to the surrounding changes.

In this Special Issue, we have collected the most recent advances in smart materials and structures, including seven original research papers and three review articles, co-authored by 65 scientists and engineers from 18 institutions and 3 industries. The research topics mainly cover advanced materials, applications of smart materials and structures, and recent development in sensing techniques.

Advanced materials and structures have attracted growing interest for their design and manufacturing flexibilities, high specific strength, and stiffness, which are superior in terms of reducing structural weight and functional complexities. Smart morphing composite technologies are developed to design and manufacture structures that can sense and respond to ambient environmental changes. Wang et al. [1] focus on reviewing recent progress in biomimetic Venus flytrap structures based on smart composites. The biomechanics of real Venus flytraps was first introduced to reveal the underlying mechanisms. Smart composite technology was then discussed by covering mainly the principles and driving mechanics of various bistable composite structures, followed by research progress on the smart composite-based biomimetic flytrap structures, concentrating mainly on the bionic strategies in terms of sensing, responding, and actuation, as well as the rapid snap-trapping process, aiming to enrich the diversities and reveal the fundamentals in order to further advance the multidisciplinary science and technological development into composite bionics.

Guo et al. [2] investigated the microstructural and mechanical performance of the Co_32_Cr_28_Ni_32·94_Al_4·06_Ti_3_ high-entropy alloy. It was found that the alloy had a single-phase, disordered, face-centered, cubic solid-solution structure and was strengthened through solid solution. Furthermore, cholesteric liquid crystals (CLCs) are molecules that can self-assemble into helicoidal superstructures exhibiting circularly polarized reflection. This is promising for an array of industrial applications, including reflective displays, tunable mirror-less lasers, optical storage, tunable color filters, and smart windows. Lee et al. [3] present a review on the electro-optic response of polymer-stabilized CLCs, i.e., PSCLCs. They exhibited dynamic optical responses that can be induced by external stimuli, including electric fields, heat, and light. Their multiple electro-optic responses and potential mechanisms were discussed in great detail.

For their unique responsive performance, smart materials and structures have been applied to reduce impact damages and improve aeroelastic stability. The vibration and impact of a humanoid bipedal robot during movements such as walking, running, and jumping may cause potential damage to the robot’s mechanical joints and electrical systems. Huang et al. [4] developed a composite bidirectional vibration isolator based on magnetorheological elastomer (MRE) for the cushioning and damping of a humanoid bipedal robot under foot contact forces. The vibration isolation performance of the vibration isolator was tested experimentally, and a vibration isolator dynamics model was developed. The MRE vibration isolator hardware-in-the-loop-simulation experiment platform based on dSPACE was built to verify the vibration reduction control effect of the fuzzy PID algorithm. It was found that the vibration amplitude was significantly attenuated, which verifies the effectiveness of the fuzzy PID damping control algorithm.

Liu et al. [5] applied topologically optimized piezoelectric smart material to improve the aeroelastic stability of bladed disks. Piezoelectric materials were embedded or bonded to each blade and use different shunt capacitance on each blade as the source of mistuning. When the shunt capacitance varies from zero (open-circuit, OC) to infinity (short-circuit, SC), the stiffness of each blade changes within a relatively small interval. In this way, the required small difference of stiffness among blades can be altered into a relatively larger difference of the shunt capacitance.

Structural health monitoring plays a vital role in determining the structural integrity of advanced materials and structures. Perfetto et al. [6] proposed a guided wave-based artificial neural network (ANN) to determine the positions of damages. The ANN was developed using FE model and trained on an aluminum plate, which was subsequently verified in a composite plate, as well as under different damage configurations. The ANN allowed detection and localization of damages with high accuracy.

Smart materials and structures are often applied in sensing techniques. Recent advances in terms of textile-based sensors, stretchable sensors, and multifunctional sensors are leading the technological development in many industries. Textile-based sensors have drawn great interest since they are superior in terms of flexibility, comfort, low cost, and wearability. They are often tied to certain parts of the human body to collect mechanical, physical, and chemical stimuli to identify and record human health and exercise. Zhou et al. [7] reviewed the recent advances in the textile-based mechanical sensors (TMSs), where sensing mechanisms, textile structure, and novel fabrication strategies for implementing TMSs were summarized. The critical performance criteria such as sensitivity, response range, response time, and stability were also investigated. Finally, the challenges and prospects were proposed in order to provide meaningful guidelines and directions for flexible sensing techniques.

As an indispensable part of wearable devices and mechanical arms, stretchable conductors have received extensive attention in recent years. The design of a high-dynamic-stability, stretchable conductor is the key technology to ensure the normal transmission of electrical signals and electrical energy of wearable devices under large mechanical deformation. Liu et al. [8] designed a stretchable conductor with a linear bunch structure and prepared by combining numerical modeling and simulation with 3D printing technology. The stretchable conductor consists of a 3D-printed bunch-structured equiwall elastic insulating resin tube and internally filled free-deformable liquid metal, showing good mechanical and electrical properties with great application potential.

Martowicz et al. [9] developed a novel measurement approach to determine the thermomechanical properties of a gas foil bearing using a specialized sensing foil made of Inconel alloy. The strain and temperature distributions were identified based on the readings from the strain gauges and integrated thermocouples mounted on the top foil. The measurements’ results were obtained for experiments that represent the arbitrarily selected operational conditions of the tested bearing. Specifically, the considered measurement scenario relates to the operation at a nominal rotational speed, i.e., during the stable process, as well as the run-up and run-out stages.

The measurement of pH has received great attention in diverse fields, such as clinical diagnostics, environmental protection, and food safety. Optical fiber sensors are widely used for pH sensing because of their great advantages. Long et al. [10] fabricated and studied a rapid-response and wide-range pH sensor enabled by a self-assembled functional polyaniline/polyacrylic acid (PAni/PAA) layer on no-core fiber. The developed sensor is high in sensitivity and quick in both response and recovery time, which is promising for detecting pH in the liquid phase with temperature variation.

We hope that this Special Issue will contribute to disseminating the latest progress in smart materials and structures, as well as stimulating the interest of its audiences to work in this important and vibrant area to promote and benefit the multidisciplinary scientific communities. Owing to the word limit on this Editorial, audiences are recommended to refer to the original papers for further information on their specific interests.
